# Cell types as species: Exploring a metaphor

**DOI:** 10.3389/fpls.2022.868565

**Published:** 2022-08-22

**Authors:** Jeff J. Doyle

**Affiliations:** Section of Plant Biology and Section of Plant Breeding and Genetics, School of Integrative Plant Science, Cornell University, Ithaca, NY, United States

**Keywords:** cell type, cell state, cell lineage, phylogeny, single cell transcriptomics, species concepts

## Abstract

The concept of “cell type,” though fundamental to cell biology, is controversial. Cells have historically been classified into types based on morphology, physiology, or location. More recently, single cell transcriptomic studies have revealed fine-scale differences among cells with similar gross phenotypes. Transcriptomic snapshots of cells at various stages of differentiation, and of cells under different physiological conditions, have shown that in many cases variation is more continuous than discrete, raising questions about the relationship between cell type and cell state. Some researchers have rejected the notion of fixed types altogether. Throughout the history of discussions on cell type, cell biologists have compared the problem of defining cell type with the interminable and often contentious debate over the definition of arguably the most important concept in systematics and evolutionary biology, “species.” In the last decades, systematics, like cell biology, has been transformed by the increasing availability of molecular data, and the fine-grained resolution of genetic relationships have generated new ideas about how that variation should be classified. There are numerous parallels between the two fields that make exploration of the “cell types as species” metaphor timely. These parallels begin with philosophy, with discussion of both cell types and species as being either individuals, groups, or something in between (e.g., homeostatic property clusters). In each field there are various different types of lineages that form trees or networks that can (and in some cases do) provide criteria for grouping. Developing and refining models for evolutionary divergence of species and for cell type differentiation are parallel goals of the two fields. The goal of this essay is to highlight such parallels with the hope of inspiring biologists in both fields to look for new solutions to similar problems outside of their own field.

“Who … has not felt the agonizing mental tension engendered by the difficulty of finding adequate verbal expression for something which has seemed to be tolerably clear in thought? And who, in such a predicament, has not eagerly welcomed the timely arrival of a suggestive metaphor … ? Such indeed is the relief, that the mind is lulled into complacency and no longer feels the urge to undertake the laborious analysis which is necessary if the makeshift metaphor is to be replaced by a direct statement in genuinely biological terms.”

J.H. Woodger. *On biological transformations*. W.E. Le Gros Clark, P.B. Medewar (Eds.), Essays on growth and form presented to D’Arcy Wentworth Thompson, Clarendon Press, Oxford (1945), pp. 95–120

## Introduction

Robert Hooke first described the cell in 1665, revealing a microscopic world of seemingly limitless variation within and among plants, animals, fungi, unicellular eukaryotes, Archaebacteria, and Eubacteria. In any one organism this variation exists as a much smaller number of classes, and because the form of a cell is coupled closely with its function, classifying eukaryotic cells into “types” has long been a goal of cell biologists (Trapnell, 2015; Miao et al., 2020). Yet to this day cell biologists do not agree on what constitutes a cell type, or even whether cell types exist at all (Clevers et al., 2017). Traditional definitions based on morphology, location, or physiology have been augmented by the unprecedented detail of single cell -omics data, particularly single cell RNA-sequencing (scRNA-seq). In the rich hyperdimensional transcriptomic space, variation has been observed among cells previously thought to comprise a single cell type … but do these represent novel cell types or are they developmental or physiological states of a known type? And how are cell types related across species?

The problem of defining “cell type” and of classifying cell types within and among organisms has been compared to the even older problem of defining “species,” philosophical elements of which can be traced to Aristotle (Ereshefsky, 2007; Shanker et al., 2017). Notably, at the dawn of the molecular biology revolution several cell biologists wrote detailed papers that drew explicitly on the philosophy and practice of systematics, applying principles from the species debate to identify and classify cell types based on the available morphological and physiological characters then available (Tyner, 1975; Rowe and Stone, 1977; Rodieck and Brening, 1983). Their conclusions, particularly concerning the amazing diversity of neurons, are now being revisited in light of new data, and their successors are again looking to the long debate on defining species with either hope or despair in the search for a single unifying definition of “cell type” (Clevers et al., 2017; Zeng and Sanes, 2017; Tasic, 2018; Northcutt et al., 2019; Xia and Yanai, 2019; Weinreb and Klein, 2020; Osumi-Sutherland et al., 2021; Xu et al., 2021). For example, the section of the paper by Zeng and Sanes (2017) on “Neuronal Cell Types as Species” begins:

In thinking about how to address the complexity of neuronal types, it may be useful to consult a field that groups individuals into types as its main preoccupation. In the field known as taxonomy, systematics or cladistics, the smallest discrete unit is the species. Although debates continue about how to define species and even whether they exist, systematics has nonetheless been a successful enterprise. The problems of defining species and neuronal cell types are similar in many ways (Tyner, 1975; Rowe and Stone, 1977; Rodieck and Brening, 1983), suggesting that there may be lessons to learn from the systematists.

It is useful at the beginning of this essay to clarify some of the terms used by Zeng and Sanes (2017). Systematics is “the scientific study of the kinds and diversity of organisms and of any and all relationships among them” (Simpson, 1961). The taxonomic objective of systematics is to classify the diversity of life, both extant and fossil, into units—taxa—that in the Linnean convention are ranked (e.g., species, genus, family), and the nomenclatural service of systematics is to provide names for these taxa. The species is considered the fundamental taxonomic unit, and therefore much effort has been spent on developing species concepts—theories of the fundamental properties of this basic organismal unit—and criteria for distinguishing them. The principal “relationships” on which systematists focus are evolutionary; the missions of systematics include reconstructing the *pattern* of evolution and understanding the *processes* that produced those patterns. Opinions differ as to whether systematics, taxonomy, or evolutionary biology is the most inclusive of these three terms. Cladistics, on the other hand, is a particular school of theory and practice within systematics/taxonomy.

During the last 40 years, much of the focus of systematics shifted to phylogeny reconstruction, which quickly became dominated by molecular rather than morphological data. In morphological phylogenetic studies the units of analysis (operational taxonomic units; OTUs) are species or higher categories, and character values are typically summarized from the variation observed across many individuals representing that OTU. In contrast, the fundamental data for molecular phylogenies are DNA sequences obtained from individual organisms (Freudenstein et al., 2016). The resulting focus on individual variation raised awareness of how population-level phenomena shape the phylogenies of genes, which has led to a paradigm shift in how variation at dozens to thousands of genes analyzed in phylogenomic analyses should be used to reconstruct organismal phylogenies. This, in turn, generated new questions about how the lineages reconstructed in such analyses align with species (Edwards, 2009; Bravo et al., 2019). During this period of revolution in data generation and analysis species concepts continued to proliferate: There were already over 20 by the end of the 1990s (Mayden, 1997), and by one count there were 34 two decades later (Zachos, 2018); a sampling of key species concepts is given in Table 1.

**TABLE 1:** Some species concepts listed by Mayden (1997) and de Queiroz (2007).

Concept	Properties	Comments
Biological Species Concept	A species comprises actually or potentially interbreeding individuals, reproductively isolated from other species	Although not discussed here, one of the most widely invoked concepts, particularly in the evolutionary biology literature
Evolutionary Species Concept	Lineages with unique roles or functions	Currently widely considered as underpinning molecular lineage-based species recognition approaches
Ecological Species Concept	Lineages occupy unique ecological niches	
Phylogenetic Species Concept	Various interpretations, including extinction of founder species when daughter species are formed (Hennigian model); Monophyletic Species Concept; Diagnosable Species Concept (within species relationships are tokogenetic, not phylogenetic)	All are in some form “cladistic”
Phenetic Species Concept	Species are recognized by clustering, based on quantitative rather than qualitative differences	Highly operational, with little explicit theoretical basis

**FIGURE 1:**
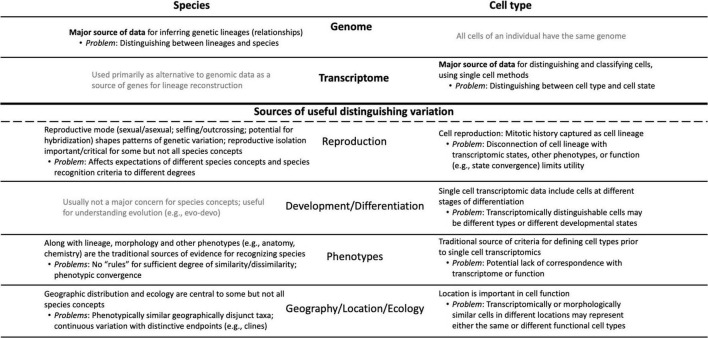
Conceptual diagram showing the parallels and differences that characterize the “cell types as species” metaphor; it is designed both as an overview and as a complement to the sections of the essay. The major sources of relevant information are listed at the top of the diagram: genomic data for species vs. transcriptomic data for cell types. Below the heavy line are the sources of data that have (in black) or have not (in gray) been used by systematists in species concepts and their parallels in cell biology. The major problems associated with each data type and source of variation are noted.

Both cell biologists and systematists are faced with the same basic problem of recognizing patterns in nature and defining the units that comprise those patterns. Both fields therefore face issues ranging from the philosophical (Do species or cell types exist, and if so what is their nature?) to the practical (What criteria should be used to recognize and classify species or cell types?). Both fields increasingly analyze individuals—organisms or cells—as their primary source of data. Of course, cell types are not species, so there are fundamental differences between them, notably the fact that genomes differ between individuals of different species, whereas every cell type of an organism has the same genome (Figure 1). However, many of the sources of variation that play roles in species concepts (Table 1) have analogs in cell biology, and in several cases pose parallel problems (Figure 1).

There is an additional parallel. Although there are systematists and cell biologists who subscribe to the nominalist position that species or cell types are nothing more than human constructs imposed arbitrarily on continuous natural variation, both categories play useful—many would say indispensable—roles in their respective fields (e.g., Garnett and Christidis, 2017; Cembrowski and Menon, 2018; Patiño et al., 2022). As one contributor to a recent compilation of cell type definitions (Clevers et al., 2017; Allon Klein) noted, “The concept of ‘cell type’ is poorly defined and incredibly useful.” Consequently, empirical practitioners in both fields, needing to interpret ever more sophisticated and voluminous datasets, press forward, defining these terms as needed for their purposes, informed to varying degrees by theoretical and philosophical debates, and guided in practice by field-wide standards enforced by reviewers, editors, and grant panels.

This essay is written from the perspective of a systematist whose studies of both evolutionary pattern and process have focused on whole genome duplication (polyploidy), a phenomenon particularly common in flowering plants (One Thousand Plant Transcriptomes Initiative, 2019) that is also a speciation mechanism and has been linked to their evolutionary success (Scarpino et al., 2014; Simonin and Roddy, 2018). The many morphological, anatomical, biochemical, physiological, and ecological effects of genome doubling have long been assumed to begin with changes at the cellular level, notably increase in cell size (Muntzing, 1936; te Beest et al., 2011). The response of cells to polyploidy is not uniform within an individual (Katagiri et al., 2016; Doyle and Coate, 2019). To understand why this should be true—a major question in plant cell biology (Roeder et al., 2021)—requires a definition of “cell type.” It was shocking to learn that there is no single definition and that cell biologists are dealing with their own “species problem.” As a practicing systematist with a longstanding interest in the issue of how molecular variation relates to species relationships (Doyle, 1992, 1997, 2021), it was apparent that the “cell types as species” metaphor had not been updated to include many developments in the ongoing species debate, particularly those involving molecular phylogenomics.

Have an additional 40 years of thinking about species, particularly based on the availability of detailed information about individual genetic lineages, produced ideas relevant to thinking about cell types? What lessons can the cell biology community learn from the species debate itself, and vice versa? Here I update the exploration of the “cell types as species” metaphor, highlighting parallels and key differences (Figure 1) and discussing some topics that could potentially cross-fertilize thinking in these two different fields. I will focus on the problem of defining cell types within an individual organism as being the most relevant comparison with the species problem. The additional dimension of understanding how cell types evolve phylogenetically (Arendt et al., 2016; Tosches et al., 2018; Shafer, 2019; Tarashansky et al., 2021; Babonis et al., 2022; Crow et al., 2022) requires definitions of both species and cell types, and a full discussion of this fascinating and critical topic is thus beyond the scope of this essay.

## Philosophical underpinnings

### Species

Ghiselin (1974) noted that “The species problem has to do with biology, but it is fundamentally a philosophical problem.” According to Shanker et al. (2017), “The earliest documented effort at a systematic classification of natural objects in ‘Western science’ is Aristotle’s principle of logical division, where every object (living or nonliving) was classified through a series of binary steps.” To Aristotle, objects can be classified because they have “essences”—properties that make them what they are, and those properties must therefore be shared by every member of the group to which they are assigned. Ghiselin (1974) continued, “… someone trained in logic should, one might think, long ago have stepped in and cleared up the confusion. Such is demonstrably not the case.” He proposed to do so by a “radical solution to the species problem”: that species should be considered individuals. Individuals do not have an essence: An individual’s parts (“members”) do not possess the same characteristics. A liver is not a brain; a leaf is not a root. So, too, the members of a species are not identical, and all need not possess the attributes that are typical of the species. Both Ghiselin (1974) and another philosopher of science, Hull (1976), contrasted species with chemical elements—Hull (1976) stated that, unlike species, “slots in the periodic table remain forever open” because “Any atom which arises with the appropriate atomic number counts as an instance of that element regardless of how, where, or when it arose.” Since the time of Darwin, however, species have been understood to be evolving lineages, and this is inconsistent with essentialism (Hull, 1976; de Queiroz, 1998; Hey, 2001). Hamilton (2012) argued that this “individuality thesis” was already central to the species concept of Hennig (1966), the founder of phylogenetic systematics (“cladistics”).

The idea of species as individuals is now widely accepted among systematists, and is consistent with most species concepts, though not with the Phenetic Species Concept, which recognizes species by overall similarity at many traits and is considered essentialist in that all organisms sharing identical characteristics would be grouped together into a phenetic species, even if they should originate convergently on a different planet (Ghiselin, 1974). There is, however, a view that species are metaphysically neither individuals nor groups, but have some elements of both, and that they are best treated as Homeostatic Property Clusters (HPCs); HPCs are marked by a set of characteristics, all of which need not be shared by all members, whose statistical correlation is due to an underlying homeostatic mechanism (Shanker et al., 2017; Casetta and Vecchi, 2021). Casetta and Vecchi (2021) pointed out that even Hull, in his 1976 “species as individuals” paper, wondered if the distinction between individuals and kinds was too crude; they noted that the cluster character criterion of HPCs avoids the problem that all members of a group defined by its essential characters must possess all of those characters, and pointed to genetic coherence as the homeostatic mechanism underlying the correlation of clustered characters. Thus, the philosophical debate continues, and yet another alternative was suggested by Shanker et al. (2017), who recommended a fuzzy set theory approach to defining species, in which different populations have varying probabilities of belonging to one or more groups.

### Cell types

Authors in the 1970s and 1980s referenced philosophical parallels between species and cell types, notably the problems with essentialist approaches (Rowe and Stone, 1977; Rodieck and Brening, 1983), but did not cite what became cornerstone literature in systematics concerning species as individuals (Ghiselin, 1974; Hull, 1976). Recently, Xia and Yanai (2019) and Moroz (2021) independently analogized cell types with chemical elements in the periodic table—the primary example of essentialist categories cited by Ghiselin (1974) and Hull (1976). Slater (2013), in discussing the philosophy underlying the definition of cell types, argued that essentialist definitions of cell type fail to meet three key criteria: that the defining properties be intrinsic; that all members and only members of the type possess those properties; and that the “essence” explain why all members also possess additional qualities in common. However, he also argued that cells comprising a cell type lack the spatiotemporal relationship to one another that is the major justification for viewing species as individuals (Slater, 2013). Instead, Slater (2013) argued that cell types are described best by a variant of the HPC concept, because they have metaphysical features of both individuals and kinds. In another parallel with species, Battaglia et al. (2013) suggested, in a paper that does not discuss philosophical issues, that cell types are treated best as fuzzy sets, in which individual cells have a probability of belonging to any of several well-defined archetypes. This seems consistent with the observation that “at the most fundamental level, single-cell dynamics is probabilistic” (Teschendorff and Feinberg, 2021); fuzzy clustering is also mentioned by Yuste et al. (2020).

### Synthesis and questions

Given the apparently innate human desire—perhaps “compulsion” would be a better word—to classify and to name, it is not surprising that there should be parallels between systematics and cell biology. This is particularly true because the philosophical options have generally been portrayed as binary, with essentialism losing out. But consider the following quote from the journal *Biology and Philosophy* (Williams, 2018):

Philosophical consensus is a rarity, and yet we may be approaching one in the philosophy of biology, and perhaps in the philosophy of science more generally, regarding the metaphysics of natural kinds. Neighborly squabbles persist, but there is widespread agreement that, for many natural kinds, their metaphysics is best understood in terms of the homeostatic property cluster (HPC) theory of kinds, or a nearby relative.

If a philosophical consensus that species and cell types are HPCs develops, might this influence theory and practice in both systematics and cell biology?

## Concepts vs. criteria

### Species

**FIGURE 2:**
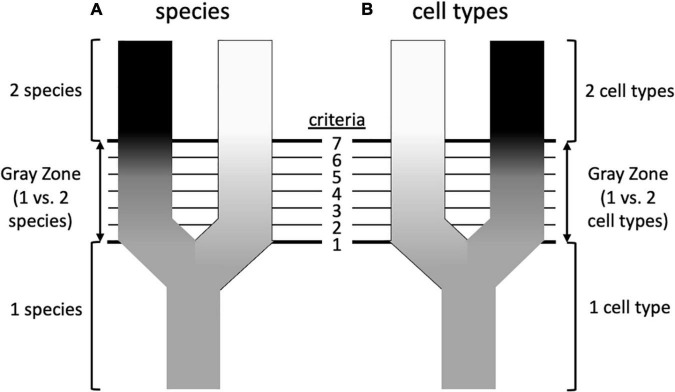
Possible similarities between speciation and the formation of cell types (based on de Queiroz, 2007). **(A)** Speciation involves the splitting of an ancestral species into two sister species. Initially, all systematists, regardless of their species concept, will agree that there is only a single species, and at some later point will again agree that there are unambiguously two species. However, in between there is a “gray zone” where there is disagreement. This is because there are various properties (an arbitrary number of seven is shown here) that diverging taxa acquire over time, such as reproductive isolation, morphological differentiation, occupation of different ecological niches, reciprocal monophyly, etc. These various properties are criteria by which systematists diagnose species, but each is prioritized differently in different species concepts. This leads to disagreement about species status in the gray zone: a systematist prioritizing criterion 1 (e.g., phenotypic differentiation) will recognize two species at an earlier stage of differentiation than a systematist prioritizing criterion 7 (e.g., reproductive isolation), which in the sequence shown here is not reached until later. This would be less of a problem if the criteria considered diagnostic always evolved in the same sequence, but this is not the case—in this example, reproductive isolation may precede morphological differentiation in other taxa. **(B)** Could a similar diagram be drawn for cell types? In this case the criteria could be such features as cell morphology, cell physiology, location in a particular organ or tissue, transcriptome, preoteome, chromatin structure, and others. To the extent that there is discordance in these features, could this underlie disagreements about whether one vs. two cell types should be recognized, or perhaps two cell types vs. one cell type with two states? The criteria that are used to recognize species are not completely independent, despite their ability to evolve in different orders (e.g., reproductive isolation can lead to morphological divergence; morphological divergence can lead to reproductive isolation), so cellular criteria need not be totally disconnected from one another, but the order in which differentiation occurs may differ. For example, there is debate about whether chromatin structure drives transcription or vice versa (Krijger and de Laat, 2017).

The diversity of living organisms presents a pattern or organization that we understand to have been formed by evolutionary processes, for which systematists are interested in developing theories; systematists also seek useful criteria for classifying the products of those processes. Unfortunately, as Hull (1997) wrote in a paper titled “The ideal species concept—and why we can’t get it,” “Applicability and theoretical significance tend to be in opposition to each other. The more theoretically significant a concept is, the more difficult it is to apply.” A solution to this problem decoupled theory from practice, adopting the stance that although there are many competing *definitions* of species, and thus many criteria for recognizing them, there might be a primary underlying species *concept* (de Queiroz, 1998, 2007). What, exactly, that concept is remains debatable, but there is widespread agreement that it is based on genetic lineage, such as some version of Simpson’s (1951) Evolutionary Species Concept (Table 1; Mayden, 1997; Padial et al., 2010; Freudenstein et al., 2016). Speciation is a continuous process, and does not occur in discrete steps that are uniform across different taxonomic groups (Stankowski and Ravinet, 2021). de Queiroz (1998, 2007) contended that much of the confusion surrounding species stems from the fact that criteria such as reproductive behavior, ecology, or the fixation of morphological or molecular characters of populations (which are lineages at a particular point in time) are met in different species at different times and in different sequences, creating a “gray zone” between what all observers would agree is one species and what all would agree is two (Figure 2). Much of the species debate, therefore, involves arguments over the primacy of different criteria for recognizing species, rather than on the fundamental nature of what a species is. According to this view, from a philosophical standpoint species concepts are generally monistic, whereas criteria for recognizing them are pluralistic.

### Cell types

In developing a definition of neuron types, Zeng and Sanes (2017) briefly explored what they considered to be the “three general schemes for defining species” in systematics: the biological species, based on reproductive isolation; phylogenetic relationships of lineages; and “a third school of systematics, known as typological, taxonomic or phenetic systematics, which groups individuals into species according to their similarity of genotype and/or phenotype.” They dismissed the first as inapplicable, and after noting several problems with a lineage-based approach for cells, adopted a transcriptomic similarity approach as being most useful for their primary purpose, classifying cell types for cell atlases (e.g., Callaway et al., 2021). Accordingly, they defined a neuronal cell type as “a population of neurons with properties that are homogeneous within the population but differ from those of other neurons.” This operational definition is both provisional and explicitly pluralistic (Zeng and Sanes, 2017); for example, Yao et al. (2021) referred to “transcriptomic cell types” as only one of various ways that cells could be classified.

A different thread in the neuronal cell type literature is that of Arendt (2008) and Arendt et al. (2016, 2019). These authors are particularly interested in the *process* by which cell types originate and evolve across species, and they defined cell type as “a set of cells in an organism that change in evolution together, partially independent of other cells, and are evolutionarily more closely related to each other than to other cells” (Arendt et al., 2016). Zeng and Sanes (2017) cited this definition in their brief discussion of phylogenetic, lineage-based approaches, and rejected it as being impractical, because the data needed for employing it are generally unavailable. Instead, they argued, “It may be more realistic to find ways to classify types within a species and then use that classification to launch an evolutionary inquiry.” In other words, although they are certainly interested in evolution, their approach prioritized *pattern* over evolutionary *process*. For their part, Arendt et al. (2019) rejected the Zeng and Sanes (2017) definition even as an operational one, because they believed that although such approaches “can provide useful classifications for neuron types within one organism, they are problematic for comparing across species. In particular, phenotypic definitions fail to distinguish two key types of evolutionary changes: the phenotypic alteration of the same cell type existing in two compared species and the origination of entirely new cell types”—precisely the process-oriented questions of greatest interest to these authors. Instead, they proposed their own operational definition, discussed below under the heading of “Phenetics and cladistics.”

### Synthesis and questions

The tension between interest in pattern vs. process, emphasizing diagnosis and theory, respectively, exists for both species and cell types. Might wider recognition of this tension by cell biologists bring these two approaches into harmony as has been done at least to some degree in systematics? As with species, different attributes of cells can be distinguished—morphology, physiology, gene expression—and it has long been known that these can be discordant (Tyner, 1975; Vickaryous and Hall, 2006). Other species concepts and criteria beyond the few cited by Zeng and Sanes (2017; Table 1) may be useful to consider. Could the insight that the order in which different attributes arise varies in the evolution of different species be applied to the order in which transcriptomes and other phenotypes appear during the development of different cell types (Figure 2)? Is there perhaps a single underlying theoretical basis for recognizing cell type, comparable to the role genetic lineage plays for many species concepts?

## Lineage

### Species

**FIGURE 3:**
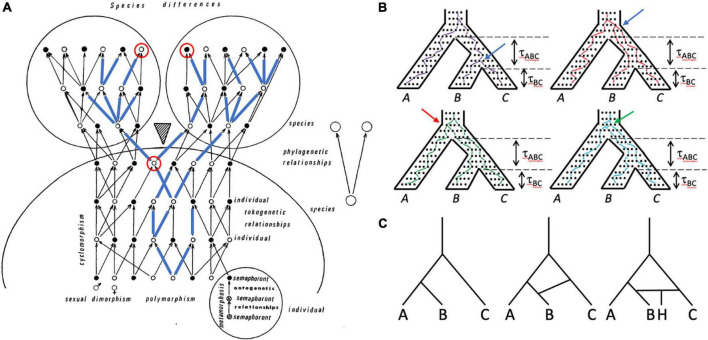
Lineages of various kinds are important in systematics and can be included within other lineages. **(A)** Tokogeny and phylogeny (modified from Hennig, 1966). A cladogenic event (shaded triangle) results in the division of a founder species into two sister species. The phylogenetic relationships of the two species are shown in the simple diagram on the right. The larger diagram shows the complexity of reticulate (tokogenetic) relationships of individuals within these polymorphic sexually reproducing species with dimorphic male (black dots) and female (white dots) individuals. Mature individuals are shown, each of which underwent metamorphosis, and thus progressed through several morphologically different character-bearing stages (semaphorants: bottom right circle), which could also provide characters for reconstructing relationships. Cyclomorphism = seasonal variation of individuals, again potentially providing characters if comparable semaphorant stages are sampled. A maternally transmitted mitochondrial DNA lineage is shown in blue lines superimposed on the arrows showing genealogical relationships. Note that although one species is fixed for this mitochondrial lineage, the other species is polymorphic for it, such that some individuals in that species possess mitochondrial genomes that are more closely related to mtDNA in the other species than to mtDNA of individuals in their own species. The mitochondrial genomes of this lineage may not be identical—they can accumulate mutations over time. Looking backward in time from the present (top of diagram), pairs of mitochondrial genomes coalesce at their most recent common ancestor. An example is shown with the two red-circled individuals, one from each species, whose mitochondrial genomes coalesce in the earlier circled individual prior to species divergence. **(B)** Gene trees are embedded within the species tree, and are shaped by the species history, but gene trees can differ from the species tree both in branch length and topology. The tree for three species with topology (A(B,C)) is shown four times, with individual neutrally evolving alleles shown as dots within it. One allele from each species is tracked backward in time from the present (bottom), with lines randomly connecting alleles in each generation and coalescing with alleles from other species until the common ancestor is reached at the top of the species tree. Time (*t*) in coalescent units (time in generations divided by effective population size) is shown for the two speciation events. Top left: purple lines track an allele coalescent history that closely tracks the species tree, having the same topology (A,(B,C)) and similar divergence times. Top right: red lines track a coalescent history that produces a gene tree topology again identical to that of the species tree, but in which alleles from species B and C coalesce much deeper in the gene tree (compare position of blue arrow in the two trees), which would suggest a much older divergence of species B and C. Bottom left: green lines connect alleles that coalesce to produce a gene tree with topology ((A,B)C), which is incongruent with the species tree; the red arrow shows the coalescence of the species B allele with the species A allele rather than with the species C allele, as in the “purple” gene tree). Bottom right: blue lines connect alleles that coalesce to produce a gene tree with topology (B(A,C)), which again is incongruent with the species tree; the green arrow points to the coalescence of the C allele with the A allele rather than with the B allele. All of the gene trees except the purple tree show deep coalescence of alleles, which in the green and blue trees creates incongruence with the species tree topology through the phenomenon of incomplete lineage sorting (ILS). Tree A has the probability 1–e*^tABC^*, whereas each of the other trees has the probability 1/3e*^tABC^*. The probability of inferring the correct species tree from trees from individual genetic loci is dependent on t, and thus on effective population size (small populations harbor fewer alleles and afford less opportunity for deep coalescence and ILS) and time (large tABC allows genetic drift to remove variation from the population, minimizing the chance of deep coalescence and ILS). The dependence of gene tree topologies and branch lengths on species history and demography is what allows species histories to be inferred from a sample of gene trees under the multispecies coalescent (MSC). **(C)** A simple three species phylogeny (left-hand tree) is complicated by introgression or horizontal transfer between species C and D (center tree), and the formation of a hybrid species (H) between species C and D (right-hand tree).

Lineages of genes, individuals, populations, and species all are important in systematics and evolutionary biology, and have complex relationships to one another (Figure 3). Despite, or perhaps because of, the central role organismal lineage plays in the species debate, the precise definition varies (Freudenstein et al., 2016); a common one is that of Simpson (1961): an ancestor-descendant series. If, as is thought, all living organisms have a single evolutionary origin, then all lineages trace back to this ancestor and all individuals are members of a single clade (an ancestor and all of its descendants) and thus are related to all other individuals to varying degrees. How should individuals be grouped meaningfully? The overall structure (topology) of this comprehensive clade of organisms is visualized differently by systematists working on different groups of organisms. Those who study multicellular eukaryotes generally refer to it as the “Tree of Life,” despite the common occurrence of hybridization and introgression in many groups, notably plants (Mallet et al., 2016), which create reticulate, non-treelike patterns (networks; Figure 3C). Paleontologists add a temporal dimension to the problem by including fossils and explicitly considering extinction (Marshall, 2017). Systematists who study unicellular organisms are necessarily conscious of reticulate relationships, to the extent that if there is a tree at all (O’Malley et al., 2010), the overall picture is that of a “cobweb of life” in which many limbs are connected by extensive horizontal transfer (Ge et al., 2005). The genealogical relationships of individuals in sexually reproducing species are also fundamentally reticulate—tokogenetic as opposed to phylogenetic (Figure 3A); such species comprise one or more lineages and represent spatiotemporally limited segments of an overall metapopulation consisting of geographically separate but genetically connected Mendelian populations. Species of sexually reproducing organisms reside at the boundary between tokogeny and phylogeny (Figure 3A).

Evolution occurs by a combination of mutation, recombination, natural selection, and genetic drift; drift is a particularly powerful force in multicellular eukaryotes, which typically have small effective population sizes, and provides a neutral explanation for many phenotypes, including cellular ones (Lynch, 2007, 2018, 2020; Lynch and Trickovic, 2020). Divergence of lineages occurs in response to both abiotic and biotic forces that restrict or promote genetic exchange. Evolution operates at the level of individuals within populations, and the results are seen in the structure of individual genomes. Gene lineages are embedded within organismal histories—the species tree shapes the gene trees of its individual members, and gene lineage phylogenies can be discordant with the organismal phylogeny (Figure 3B; Maddison, 1997; Degnan and Rosenberg, 2006). With assumptions of neutral evolution and minimal gene flow between the units of evolution (species, generally as defined in the Evolutionary Species Concept), the multispecies coalescent (MSC; Rosenberg and Nordborg, 2002; Kubatko, 2019) has been widely adopted from population genetics as a unifying statistical model for studying the pattern and process of species divergence, revolutionizing the way its adherents use molecular data to reconstruct phylogenies (Bravo et al., 2019). Moreover, as Kubatko (2019) has pointed out, “Within the last decade, methods for species delimitation have increasingly cast the problem in the framework of the multispecies coalescent.” But this has sparked debate about whether what is recognized by MSC-based delimitation methods are “real” species as opposed to simply “lineages” that comprise portions of species (Jackson et al., 2017; Sukumaran and Knowles, 2017; Leaché et al., 2018; Chambers and Hillis, 2019; Sukumaran et al., 2021). Debate about the relationships among lineages, populations, and species, involves models of speciation … which in turn requires a definition of “species.”

### Cell types

**FIGURE 4:**
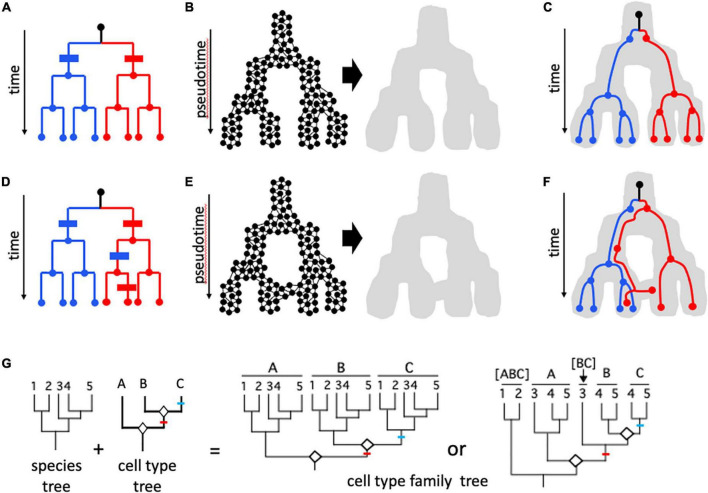
Cell lineages. **(A–F)** Cell mitotic lineages vs. transcriptomic manifolds (Wagner and Klein, 2020). **(A,D)** Tree-like relationships describing clonal cell lineages progressing in real time from a founder cell (black dot at top of dendrogram) to eight cells in red and blue sub-lineages through a series of mitotic cell divisions. **(B,E)** Construction of state manifolds from single cell transcriptomic data. This involves aggregating cells with similar transcriptomic positioning in high-dimensional parameter space (e.g., a UMAP projection) to produce a landscape/manifold (gray shape) which reflects gene expression dynamics in pseudotime. Manifolds can be non-reticulate (acyclic) and tree-like as in **(B)**, or can form reticulate (cyclic) networks as in **(E)**. In **(C,F)**, cell mitotic lineages from **(A,D)**, respectively, are shown included within the developmental manifold. In **(C)**, there is congruence between the topologies of the lineage and the manifold: transcriptomic signatures are perfectly correlated with cell lineage, and this can be shown in panel **(A)** as two synapomorphies (blue and red bars) with no homoplasy (additional change in parallel). In contrast, in **(F)** there is incongruence between cell lineage and transcriptome; with information from cells at intermediate developmental stages it is possible to see that the incongruence is due to two events: an early shift from the right-hand branch represented by most “red” cells to the left-hand (blue) branch of the manifold, and a later shift of some but not all cells of this lineage back to the red fate. This is shown in **(D)** with two additional changes (apomorphies)—a blue change (parallelism) in the red lineage followed later by a reversion to the red state for one of the two cells. These two additional steps represent homoplasy in the mapping of gene expression characters on the mitotic lineage tree. **(G)** The evolution of homologous cell types, following concepts of Arendt et al. (2016, 2019). A phylogeny of five species (1–5) is shown, along with a tree relating three cell types (A–C) formed by “cell typogenesis” (white diamonds at nodes). Together, speciation and cell typogenesis produce a cell type family tree (analogous to speciation and gene duplication producing a multigene family tree). Speciation and cell typogenesis are two independent processes, and so can occur at different times relative to one another. Two scenarios are shown. In the first case, the two origins of new cell types occurred prior to the speciation events that produced the five species; the common ancestor of the five species therefore possessed all three cell types, and therefore all five species possess cell types A, B, and C; A_1–5_, B_1–5_, and C_1–5_ are analogous to orthologous genes. In the second scenario, cell typogenesis events are interspersed with speciation events: the first cell typogenesis event occurs after the ancestor of species 1 and 2 diverged from the ancestor of species 3–5, and the second cell typogenesis event occurs after the divergence of the ancestor of species 3 and the ancestor of species 4–5. Therefore, only species 4 and 5 possess all three cell types. Species 3 possesses cell type A and the progenitor of cell types B and C; species 1 and 2 possess only a single cell type, derived from the progenitor of all three other cell types. The analogous situation in a multigene family would consider [BC] in species 3 to be co-orthologous with B and C, which are in-paralogs in those two species; [ABC] to be co-orthologous with A, [BC], B, and C. One or more of cell types A, [BC], B, and C might be considered the same cell type as [ABC], depending on the amount of divergence following speciation; for example, cell type A in species 3–5 and cell type [ABC] in species 1 and 2 might retain sufficient transcriptomic similarity to be considered “the same,” with the sister cell type clade in species 3–5 differentiating transcriptomically by acquiring a modified core regulatory complex (CoRC) and new sets of effectors. Red and blue lines indicate apomorphies (novel CoRCs and apomeres) associated with new cell types B and C, respectively.

As in species biology, “lineage” has more than one meaning in describing relationships of cells to one another (Figure 4). Just as individual organisms trace their ultimate origin to the common ancestor of all life, cells of an individual multicellular organism belong to a common historical lineage, beginning with the single cell of the zygote. Cell fusion occurs (outside of fertilization) in some animal organs (e.g., skeletal muscle) but in plants does not contribute to the generation of mature cell types (Brukman et al., 2019). Thus, for the most part, the cell lineage of a multicellular eukaryote, even more than is true of organismal lineages, can be described as a tree, and such cell lineage trees can now be reconstructed with increasing precision (Wagner and Klein, 2020). Slater (2013) noted that “Unlike species, cells do not fit into a *single* phylogenetic tree. Rather, development in each organism defines its own local tree,” but although this may cause philosophical concerns, the problem is minimized if, as here, the focus is on the cells of an individual in a single species. Rodieck and Brening (1983) suggested that “It might appear that the lineage of ganglion-cell types, expressed during development, would provide a causal agent as useful as the role genealogy plays in the classification of species.” However, they noted that despite the strong superficial resemblance between lineage trees of cells and organismal phylogenies, there are fundamental differences (Rodieck and Brening, 1983). With species, a lineage (however, defined) can be related ultimately to mutational variation at the genomic level. In contrast, all cells of an individual organism possess fundamentally the same genome (ignoring somatic mutation and phenomena such as endopolyploidy), and the process of differentiation that is analogous to speciation occurs through epigenetic mechanisms (Figure 1). The immediate ancestor of a species cannot produce a species that belongs, genetically, to a different clade, but a multipotent stem cell is theoretically capable of producing any mature cell type. The connection between phenotype (the mature cell’s transcriptome, morphology, etc.) and epigenotype for cells is thus looser than a whole organism’s phenotype to genotype relationship. Rodieck and Brening (1983) concluded that “if there proves to be little or no correspondence between the lineage map and its phenotypic expression, then it is unlikely that the lineage map will become a useful and widely accepted ordering of ganglion-cell types.”

Currently, the transcriptome is the most studied “phenotypic expression” of cells and variation in the full transcriptome or in the expression of specific sets of genes is the major source of data for defining cell states and types (Almeida et al., 2021; Sacher et al., 2021); as such, it has much the same relationship to cell types as the genome does to species (Figure 1). Gene expression changes as cells differentiate, and this can be visualized as a landscape (“manifold”) in pseudotime (Figures 4A–F). Depending on the method used to create the manifold from high dimensional transcriptome data, the manifold can be an acyclic graph (tree) or cyclic graph (network, Figures 4A–F; Wagner and Klein, 2020). Mani and Tlusty (2021) simulated millions of developmental programs in model-generated “organisms” and characterized their graph topologies; surprisingly, tree-like topologies were rare, and in most cases multiple cell lineages converged on the same terminal cell type. Wagner and Klein (2020) portrayed mitotic lineages as included within expression manifolds (Figures 4C,F). Alternatively, expression fate can be shown as character changes on mitotic lineage trees; discordance between expression state and mitotic lineage then appears as convergent change or state reversals, like any character showing homoplasy on a phylogenetic tree (Figures 4A,D). The potential for discordance between phenotype (expression manifold) and cell mitotic lineage is what led both Zeng and Sanes (2017) and Arendt et al. (2016, 2019) to reject using cell lineage for defining mature cell types. For example, in blood development (hematopoiesis), different cell lineages can produce functionally similar cells (Yáñez et al., 2022). Nevertheless, transcriptomic state cannot be assumed to be more important than lineage; in hematopoiesis, Weinreb et al. (2020) found that “sister cells tended to be far more similar in their fate choice than pairs of cells with similar transcriptomes” and concluded that chromatin structure might provide information that neither lineage nor transcriptome reveal.

The concept of homologous cell type lineages (Vickaryous and Hall, 2006), as distinct from mitotic lineages or developmental lineages, has been explored and developed by Arendt et al. (2016, 2019). It transcends the individual or species and considers the evolution of cell types over phylogenetic timescales rather than individual lifetimes (Figure 4G). Cell types in this sense resemble phenotypic character trees for cladistic analysis (Pogue and Mickevich, 1990; Musser and Wagner, 2015) in being embedded in organismal phylogenies (“species trees” in population genetic terminology; e.g., Nei, 1987), but perhaps have their closest analog in gene family trees, which have complex homology and functional relationships due to duplication (e.g., Glover et al., 2019). The potential for homologous cell type lineage phylogenies to provide a unifying, objective criterion for defining cell type (Almeida et al., 2021) is discussed in more detail below.

### Synthesis and questions

The biology of both species and cells involves more than one kind of lineage, whose relationships to one another are complex, may be nested, and can be incongruent (Osumi-Sutherland et al., 2021). Homoplasy, non-homology generated by parallelism and convergence, reveals gaps in our knowledge (Nixon and Carpenter, 2012) and thus is of fundamental interest in understanding the evolutionary process (e.g., Wake et al., 2011). Similarly, exploration of the complex connections between mitotic cell lineage and cell state, often involving state convergence, is an exciting area in cell biology (Battaglia et al., 2013; Konstantinides et al., 2018; Wagner and Klein, 2020; Osumi-Sutherland et al., 2021; Yáñez et al., 2022).

Much has been accomplished in systematics by employing models of the coalescent process to infer species relationships from the gene lineages embedded in them (Figure 3B). In the coalescent approach, incongruence between two types of lineages—species and gene—is a key source of data, rather than a problem. It also can be invoked to account for phenotypic homoplasy (hemiplasy; Guerrero and Hahn, 2018). Can the cell-level models that are being developed (Weinreb et al., 2020; Teschendorff and Feinberg, 2021) in an analogous way harness the discordance between the mitotic and transcriptional lineages of cells to define cell types?

## Categories, states, and semaphorants

### Species

Accommodating variation at different organizational levels, from genes to genomes to populations, is a challenge for species definitions both in theory and in practice. Mutation, which includes not only base substitution but also insertions, deletions, transpositions, chromosome structural changes (inversions, translocations), and recombination, ensures that individuals are rarely genetically identical. An appreciation of copy number variation in multicellular eukaryotes has led to the adoption of the pangenome concept from bacterial genomics; it is now recognized that no single individual plant or animal genome provides a complete picture of gene content in its species (e.g., Gao et al., 2019; Miga and Wang, 2021).

The pangenome concept captures one aspect of genetic variation among related individuals, which is often structured at the level of populations. The potential for “over-splitting,” particularly when the distinguishing characters are microscopic or otherwise cryptic, is not a new concern, nor is it confined to any particular species concept or recognition criterion—there has always been debate between lumpers and splitters. Taxonomic ranks, both formal (subspecies) and informal (variety), have been used to designate groupings that do not rise to the level of differentiation considered to merit species status; even many systematists who hold that species are “real” natural entities consider these categories to be artificial constructs, as is also true of genera, families, and higher taxonomic ranks.

Variation also occurs over the course of development, and individuals can appear very different at different stages of their lives. An acorn does not look like the oak tree that produced it; larval and pupal stages do not look like the butterfly they will become. Yet in both cases the individual at each different stage represents a single species, and for the purposes of defining that species and reconstructing its phylogenetic relationships individuals at any life stage are “character-bearers”—what the founder of cladistics, Willi Hennig, termed “semaphorants” (Figure 3A; Havstad et al., 2015). A complete description of a species includes all of its semaphorants; species can be compared, and their phylogenies reconstructed, from any homologous characters gleaned from comparable semaphorants in different species. The importance of semaphorants diminished with the reliance on molecular data for phylogeny reconstruction, for which individuals are typically the units of gene or genome sampling (Freudenstein et al., 2016). This certainly is true in the concatenation paradigm, where the sequences of multiple genes sampled from an individual form a single row in the data matrix that is then aligned with the aggregated sequences from each other individual. However, methods based on the MSC, though they sample individuals, do so as representatives of a species (Bravo et al., 2019), so each individual is again potentially a semaphorant, bearing a subset of the characters and character states found in the species.

### Cell types

Like conspecific individuals, cells of the same type are not identical (Cembrowski and Menon, 2018; Usaj et al., 2021). At the transcriptomic level, a source of cell-to-cell variation is dropout “due to low amounts of mRNA in individual cells and inefficient mRNA capture, as well as the stochasticity of mRNA expression” (Qiu, 2020). From a practical perspective, dropout is similar to the longstanding problem of missing data in phylogenetic data matrices (Xi et al., 2015). Biologically, transcriptional bursting (Tunnacliffe and Chubb, 2020) means that the full picture of the transcriptome of a cell type cannot be obtained from any single cell, much as the pangenome of a species cannot be inferred from a single genotype. Consequently, in practice, much of “single cell” biology involves clusters of cells with similar transcriptomes or other -omic phenotypes (e.g., Coate et al., 2020); thus, Arendt et al. (2019) recommended using small groups of cells with very similar expression (“metacells”), rather than individual cells, to reconstruct cell type trees. Because technical causes of dropout are expected to be roughly the same for all cell types in a given experiment on a per cell basis, any differences in the pattern of dropout among populations of cells should be due to biological causes. Qiu (2020) found that “dropout pattern in scRNA-seq data is as informative as the quantitative expression of highly variable genes” and suggested “embracing” dropout by using binarized data rather than transcript counts instead of ignoring it. Bouland et al. (2021) found that a similar approach did indeed capture biologically meaningful variation in single cell transcriptomes. Nevertheless (Jiang et al., 2022), criticized methods that use binarized gene expression data, because they ignore information from differential expression of the same genes in different cell types.

Regardless of the lineage to which a particular cell belongs, its expression changes as it makes the transition from stem cell to its mature cell state. Single cell or single nucleus RNA-seq experiments produce a “snapshot” that includes mature cells, the stem cells destined to give rise to them, and cells in transitional states. The picture is tremendously rich in detail (Teschendorff and Feinberg, 2021), and includes information that can be used to identify the position of cells in “pseudotime” along a differentiating cell lineage (Campbell and Yau, 2018), and to predict the future states of cells using information on spliced vs. unspliced mRNA molecules (RNA velocity; La Manno et al., 2018). Nevertheless, because it is a snapshot involving multiple cells, Weinreb and Klein (2020) noted that single cell transcriptomic approaches alone cannot determine, for any particular cell, when “progenitor cells become committed to one or more fates or how many distinct paths might lead cells to the same end states.” The question of fate determination is relevant not only for defining cell type but also for such issues as determining whether plants have a committed germline (Lanfear, 2018; Burian, 2021). Sagar and Grün (2020) concluded that emerging data are “challenging the classical view of cell fate commitment as a discrete binary decision process where immature multipotent progenitors become lineage restricted in a stepwise fashion”; instead, differentiation may occur probabilistically “in a continuous transcriptional and chromatin landscape.” Other authors also have emphasized the continuous nature of cellular commitment (Xia and Yanai, 2019; Quake, 2021).

Tasic (2018) noted that whereas the cellular equivalents of phyla, such as the “cardinal classes” of neurons described by Fishell and Kepecs (2020), might be readily identifiable, the same is not true of lower hierarchical levels that correspond to genera or species. This is due to continuous variation, even within some narrowly defined cell types (Cembrowski and Menon, 2018; Efroni, 2018; Quake, 2021; Shojaee et al., 2021), and to the associated problem of distinguishing cell type from cell state. A single cell type can differ in essential characteristics over the course of its life—what is considered the same cell type can have different states due to development, environment, treatment, or location. Some of the authors of the Clevers et al. (2017) poll of definitions of cell type appear to subscribe to the nominalist position that “cell type” is an arbitrary designation, and that only cell states exist. This echoes the position of some systematists that lineages are real, whereas species are arbitrary (Vaux et al., 2016; Mishler, 2021). But most cell biologists see a real distinction between cell types, which are “hard-wired,” and cell states, which are “soft-wired” (Arendt et al., 2016, 2019; Morris, 2019). However, the distinction between hard- and soft-wired can vary over the course of a cell’s developmental trajectory (Fishell and Kepecs, 2020). Cembrowski and Menon (2018) and Tasic (2018) have suggested that cell states are reversible, whereas cell types are not, at least under standard conditions.

In the analogy of cell types with elements in the periodic table, cell states are like isotopes (Xia and Yanai, 2019; Moroz, 2021). Alternatively, extrapolating from Tasic’s (2018) comparison with taxonomic categories, if cell types are analogous to species, then cell states might be analogous to subspecies or varieties. A different approach adopted from systematics would consider cell states as semaphorants—different manifestations of the same biological entity, united by some core features but bearing a unique set of characters depending on their stage of development and physiology. But this approach requires that the entity to which semaphorants belong—species or cell types—first be defined.

### Synthesis and questions

A number of cell biologists have recognized the parallels between the issue of how broadly a cell type or a species should be defined, referring explicitly to “lumpers and splitters” (Rodieck and Brening, 1983; Armañanzas and Ascoli, 2015; Tasic, 2018; Yuste et al., 2020). Decisions about how many species to recognize are often guided by the kinds of variation available to the taxonomist, particularly whether the characters that distinguish taxa are easily discernible. For example, the Eastern spring beauty (*Claytonia virginica*) comprises several well-differentiated chemical and chromosomal lineages with distinctive geographical ranges, but morphological variation among plants from these groups is cryptic (Doyle, 1984), and therefore the groups have not been named formally. This type of subjective practical decision is specific to each organismal group, presumably because each group has a different sequence of character evolution (Figure 2; de Queiroz, 1998, 2007), and is captured at a particular time point in its evolutionary trajectory—systematists cannot see its future, and in all but rare cases lack a detailed enough fossil record to know its past.

In contrast, the snapshot available to cell biologists provides a much more comprehensive sample of differentiation, from stem cells to mature cells, for an individual at the developmental stage at which it is studied. Moreover, this process is expected to be similar across all species and at all stages in the lives of individuals. Of course, because an individual sampled at a specific stage of its life is itself a semaphorant for its species, a complete picture of cell types even of a single species may not be obtainable without greater sampling. But the questions troubling cell biologists about state vs. type seem more tractable than those involving species because there is more hope that a shared set of fundamental rules exist to be discovered. Will such rules reinforce the legitimacy of “cell type” as a theoretical concept as well as a practical category? Regardless of that answer, what characters could be used to define cell types?

## Phenetics and cladistics

### Species

The late plant taxonomist Arthur Cronquist is reputed to have said that “a good species is what a good taxonomist says it is”; Mayden (1997) calls this the Morphological Species Concept (Table 1). By a “good” taxonomist, Cronquist meant one with a keen eye, familiar enough with the taxa in question to be able to discern the key characters by which meaningful groupings of individuals could be discriminated from one another, filtering out polymorphisms, plasticity, and other uninformative variation. This subjective approach to taxonomy was challenged in the 1960s and 1970s by “numerical” taxonomists, who instead embraced variation, using large numbers of characters with minimal *a priori* filtering in “phenetic” analyses that identified clusters based on overall similarity rather than on criteria from evolutionary theory. Phenetic groupings were dependent on the algorithms used, and recognition of species under the Phenetic Species Concept was strictly operational and based on arbitrary similarity cutoffs (Mayden, 1997); the trees (dendrograms) produced were meant to portray similarity rather than genealogy or evolution. Genome clustering, also an operational approach (Hull, 1997), continues to be used in bacteria, with delimitation of species being proposed for genomes with Average Nucleotide Identity (ANI) scores >95% (Konstantinidis et al., 2006; Jain et al., 2018) on the theory that in nature there is a discontinuity in genetic variation at this level; the approach is controversial (Murray et al., 2021; Rodriguez et al., 2021).

**FIGURE 5:**
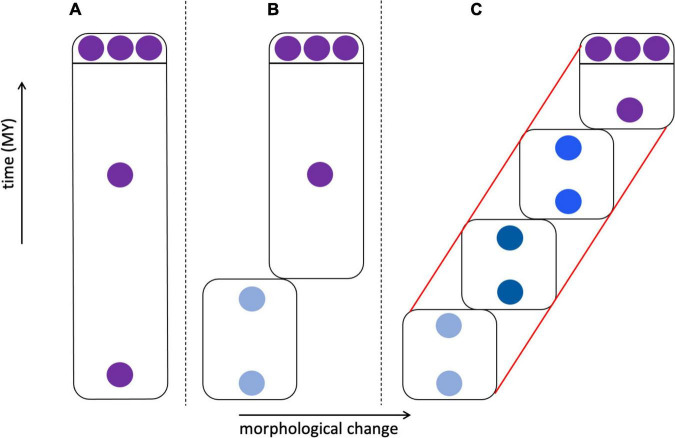
Morphological change in species through time. In **(A–C)** three individuals sampled from an extant species are shown at the top, above the line, with fossil individuals below the line; fossils are either morphologically identical to modern individuals or differ from them to varying degrees. **(A)** Fossil and modern individuals are similar enough that they are classified as the same species. **(B)** Fossils exist that are identical to modern individuals, but deeper in the fossil record these are replaced by individuals that lack apomorphic (derived) characters. This could lead to the recognition of two chronospecies, with the modern species marked by autapomorphies. **(C)** A relatively complete fossil record links early fossils through a series of transitional forms leading to the modern species with its set of autapomorphies. This could lead to the recognition of one species (red lines) showing anagenesis, four chronospecies (boxes), or some intermediate number of taxa. **(B)** vs. **(C)** represent punctuated vs. gradual speciation patterns.

The cladistic approach largely supplanted phenetics in eukaryotic systematics by the 1980s, after considerable and often vitriolic debate. The original name of cladistics—“phylogenetic systematics”—clearly rooted its approach in the evolutionary process, with the goal of identifying clades comprising ancestors and their descendants defined by shared-derived characters (synapomorphies). In cladistic analysis, the principle of parsimony is used to select among phylogenetic trees (cladograms) whose topologies depict the relationships of species and higher taxa. The use of cladistic parsimony methods for inferring organismal (as opposed to gene) relationships below the species level is more controversial and led to various versions of the Phylogenetic Species Concept (Table 1; Mayden, 1997; de Queiroz, 2007; Freudenstein et al., 2016). One version holds that phylogeny ends at the species level; cladistic approaches and terminology should not be applied to within-species tokogenetic relationships (Figure 3), and species should instead be defined by characters or combinations of characters fixed in individuals and populations. Other versions require species to be monophyletic, forming a clade in which individuals share at least one synapomorphy, so that the species is defined by at least one autapomorphy (a derived character unique to that species and not shared with other species). In paleontology, this consideration is relevant to the issue of when to recognize new species that differ from modern descendants, given an incomplete fossil record. In a model where anagenetic change occurs—change without the formation of new clades by cladogenesis, presumably due to speciation—morphological changes are presumed to be autapomorphies, and thus could mark new “chronospecies” (Figure 5; Silvestro et al., 2018; Marshall, 2019).

There is no requirement that the characters by which species are recognized be responsible for causing their divergence from their progenitor, or even that they be adaptive. However, “speciation genes” that could drive divergence, for example by leading directly to reproductive isolation (Figure 1), remain a topic of interest in the evolution literature (Burdon and Marshall, 1981; Wang and Hahn, 2018).

In the 1990s, the cladistic parsimony approach to phylogeny reconstruction was challenged by maximum likelihood and Bayesian methods employing explicit models of molecular evolution. Character change is treated probabilistically and not as a source of discrete apomorphies as in cladistics. Model-based approaches are now the mainstay of phylogenomics; like the MSC methods that underpin the species tree paradigm (Bravo et al., 2019), they are rooted in population genetics.

### Cell types

One of the Clevers et al. (2017) authors (Allon Klein) wrote, “No single attribute has served for cell type classification. Yet ‘we know it when we see it.”’ This echoes the Morphological Species Concept. But, he continued, “We are left with a functional but flawed taxonomy: functional, because it provides a language to describe biology; yet flawed, because it lacks consistency.” The search for objective criteria has led to the recognition of “transcriptomic cell types” by the neuron community (Zeng and Sanes, 2017; Cembrowski and Menon, 2018; Yuste et al., 2020; Callaway et al., 2021; Yao et al., 2021). Like phenetic clustering approaches to species recognition, this approach does not make strong theoretical claims—its primary goal is classification (Zeng and Sanes, 2017). It also has some of the same problems as phenetics. Breaking up continuous variation can involve subjective decisions (e.g., Cembrowski and Menon, 2018; Sagar and Grün, 2020), and the identification of biologically meaningful clusters from the high dimensional data produced by expression of 20,000 or more genes (Efroni, 2018; Quake, 2021) makes clustering more an art than a science (Kobak and Berens, 2019; Capdevila et al., 2021). Indeed, Chari et al. (2021) referred to single cell genomics as a “specious art” based on their finding that commonly used unsupervised clustering approaches for dimensionality reduction do a poor job of grouping adjacent cells in this complex parameter space. Northcutt et al. (2019) also noted limitations of transcriptomics but expressed optimism that the approach “can partially indicate identity, particularly once supervised methods incorporating known cell identification are employed.”

What of cladistic approaches? Vickaryous and Hall (2006) reconstructed most parsimonious trees from a matrix of 19 biochemical, physiological, and morphological characters in their study of neuron diversity. However, they did not use this approach to define cell types, but rather to apportion predetermined cell types into groups in a hierarchical classification. Raj et al. (2018) and Jones et al. (2020) also used parsimony methods for reconstructing cell lineage phylogenies, but not to identify cell types. Yuste et al. (2020) considered trees generated from (phenetic) clustering methods to be in “the historical tradition of using cladistics to classify organisms, assuming common ancestors in their evolution and synapomorphies (shared derived traits) among related clades”; this conflation of phenetic dendrograms with cladograms would horrify any cladist!

**FIGURE 6:**
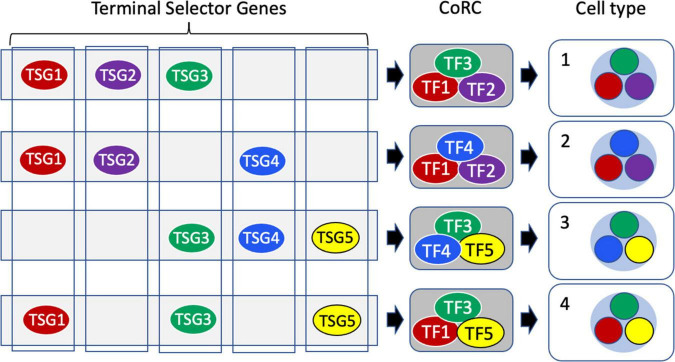
Terminal selectors, core regulatory complexes and cell type determination. Terminal Selector Genes (TSGs) encode transcription factors (TFs) each of which controls the expression of a suite of downstream effector genes. Terminal selector TF proteins act combinatorially, assembled into Core Regulatory Complexes (CoRCs) that also include accessory proteins. At the cell level, the different TFs of the CoRC each direct the transcription of their effector genes to produce a distinctive overall transcriptome characteristic of a specific cell type (nucleus shown as a light blue circle, with the sub-transcriptomes comprising expression of effectors color-coded to reflect the terminal selector TF regulating their expression.

In contrast, the sister cell type theory of Arendt (2008) and Arendt et al. (2016, 2019) is explicitly phylogenetic, driven by the concept of homology as similarity due to common descent. A cell type is defined operationally as “a set of cells accessing the same regulatory program driving differentiation” (Arendt et al., 2019). Each cell type is characterized by the presence of a unique Core Regulatory Complex (CoRC; Figure 6), defined as “A protein complex composed of terminal selector transcription factors that enables and maintains the distinct gene expression program of a cell” (Arendt et al., 2016). The concept of terminal selector genes (TSGs), as high level cooperating regulators, generally transcription factors (TFs), that act to specify and maintain cell identity by controlling downstream selector and effector genes and by repressing other identities, has been developed by Hobert (2008; 2016; 2021); Patel and Hobert (2017); Hobert and Kratsios (2019); Sun and Hobert (2021). The CoRC includes not only TSGs but also “more general cofactors” (Arendt et al., 2016). The CoRC model requires “that cells of the same type implement the same hard-wired differentiation program, using the same transcription factors, regulatory elements, microRNAs, and so on” (Arendt et al., 2019). New cell types arise from an existing cell type by modification of the CoRC, for example by duplication and divergence of TSGs. During eukaryotic diversification “cell typogenesis” led both to the evolution of new functions and the partitioning of existing functions of already complex cell types, analogous to subfunctionalization of duplicated genes (Arendt, 2008; Arendt et al., 2019).

Cell type evolution can be represented as a phylogenetic tree of homologous cell types, hierarchical in nature like a species cladogram (Figure 4G; Arendt et al., 2016, 2019). The CoRC thus corresponds to the autapomorphy that defines the monophyletic species in some versions of the Phylogenetic Species Concept (de Queiroz, 2007) and should be shared by all members of the cell type “species” regardless of their state. It should, in theory, be a better apomorphy than the cell type marker genes used in many single cell studies to identify cell clusters, particularly in cross-species comparisons (Liang et al., 2018), since it is upstream of their expression. It is also of interest that this apomorphy is causative: Its formation creates and maintains as well as defines a cell type. This distinguishes the CoRC apomorphy from the characters that define species, whose adaptive value is unknown and which often may be neutral.

There are many challenges to making the CoRC concept truly operational (Zeng and Sanes, 2017; Arendt et al., 2019), beginning with identifying CoRCs (Almeida et al., 2021). As protein complexes, CoRCs cannot be assayed simply by monitoring transcription of TF genes, since TF proteins can be transcribed in one cell type and function in others (Clark et al., 2020); advances in single cell proteomics ultimately will resolve this problem (Labib and Kelley, 2020). Xia and Yanai (2019) discussed the use of CoRC as a means of defining cell type, noting that the CoRC remains largely conceptual for most cell types, and that a proxy for the CoRC is to use expression profiles of TFs, among which are TSGs of the CoRC (see also Almeida et al., 2021). Monitoring transcription is difficult enough given that many if not most genes, including TFs, are expressed across cell types (Figure 6; Xia and Yanai, 2019; Coate et al., 2020); regulatory regions differ by the strength with which they bind TFs rather than solely by which TFs they bind (Mora-Martinez, 2021); and TF expression is quantitative, not qualitative (e.g., Fishell and Kepecs, 2020; Shojaee et al., 2021). The assembly of such complexes can also be spatially and temporally disjunct from where they function (Charest et al., 2020). At the transcriptional level, “apomeres”—modules of genes defining novel or modified functions—are the apomorphies that define a cell type (Arendt et al., 2016, 2019).

**FIGURE 7:**
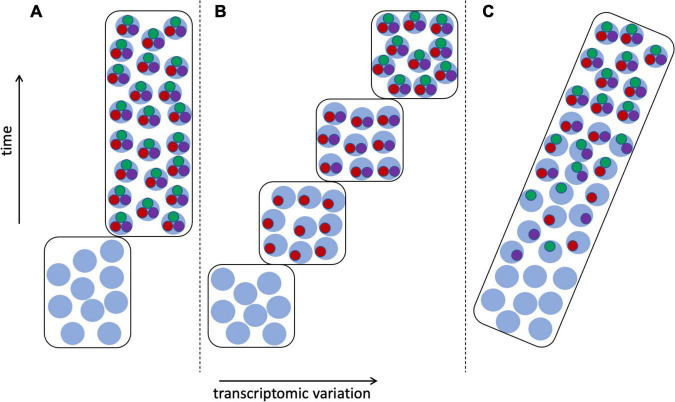
Transcriptomic variation of differentiating cells. Transcriptomes as in Figure 6, cell type 1: with stem cell transcriptomes shown as light blue circles, mature cells expressing apomeres of all three terminal selector TFs (red, green, and purple circles), and cells at intermediate stages of differentiation having 1–2 apomeres. **(A)** The CoRC defining the mature cell type is assembled in a single step, resulting in clear demarcation of two cell types. **(B)** Stepwise assembly of the CoRC, in the order TF1, TF2, and TF3, resulting in several distinguishable states, each of which could be considered a cell type. **(C)** Random assembly of the CoRC during differentiation would produce more gradual or continuous transcriptomic change from stem to mature cell type.

Arendt et al. (2019) particularly noted the problem of the continuous nature of cell type variation among developmental and physiological states, as discussed above, including such issues as delayed commitment of a cell lineage to a particular fate (Wagner and Klein, 2020; Weinreb et al., 2020). Much would seem to depend on when and how the CoRC is assembled (Sagar and Grün, 2020)—whether as a single step or as a sequence of additions, and if the latter, whether these intermediate states are functional, and how they correspond to stages of tissue, organ, or individual development (Figure 7). If intermediate stages of the CoRC are functional—as might be expected given the ability of each TS to activate its own regulatory network (Hobert, 2008)—then the question of defining cell types becomes similar to the issue of determining the boundaries of chronospecies from fossils (Figure 5). Shojaee et al. (2021) referred to cell state transitions being “choppy” rather than truly continuous, occurring in waves, echoing the punctuated equilibrium vs. gradualism debate in paleontology (Benton and Pearson, 2001). But in the case of cell types there is greater hope of resolution given the availability of a much more complete inventory of cells, including cells in the process of differentiating, in contrast to the fossil record, which is generally fragmentary and is dominated by extinction events (Benton and Pearson, 2001; Marshall, 2017).

Arendt et al. (2019) recommended the application of objective phylogenetic methods for constructing cell type phylogenies, among which they included both parsimony and distance methods. Although they also mentioned modeling the cellular evolution process, they did not mention any specific models of cell differentiation or evolution. The need to develop probabilistic models of neuron cell commitment has been noted in several papers (Sagar and Grün, 2020; Wagner and Klein, 2020; Yuste et al., 2020). Mukamel and Ngai (2019) proposed that “Computational and statistical modeling of transcriptomic measurements from a range of neuron types could indicate which transcription factors are the core regulators of cell type identity.” In contrast, Fishell and Kepecs (2020) rejected what they call the “classic view” of specification of cell type by combinatorial action of TFs in favor of an attractor model built on Waddingtonian concepts of developmental commitment (Trapnell, 2015; Efroni, 2018); they contended that “combinations of TFs can initialize but not realize cell fates.”

### Synthesis and questions

The CoRC concept has the potential to provide both a theoretical underpinning and an operational criterion for defining most if not all mature cell types in a way unthinkable for species, with their diverse modes of origin. Is the CoRC indeed a universal feature of cell biology? If so, can methods be developed to assay it as an operational criterion? Can models of cell type differentiation be developed along the lines of the model-based approaches that revolutionized systematic biology for phylogeny reconstruction, and more recently (though more controversially) for defining species? As Yuste et al. (2020) put it, “A robust statistical framework that enables a quantitative definition of cell type (or tendency to be a type) is clearly needed.” What information should such models incorporate, beyond transcriptomic data? Could chromatin criteria be incorporated (Weinreb et al., 2020; Winick-Ng et al., 2021)?

## Role and function: The ecology and geography of species and cells

### Species

Freudenstein et al. (2016) argued that the concept of lineage that increasingly has come to dominate systematics is necessary but not sufficient for defining species. They noted that the commonly invoked Evolutionary Species Concept involves more than just history in its definition. A species is “a phyletic lineage (ancestral-descendent sequence of interbreeding populations) evolving independently of others, *with its own separate and unitary evolutionary role* and tendencies” (Simpson, 1951, 1961; italics added here). Freudenstein et al. (2016) summed up their thesis (italics in original):

“We argue rather for the crucial importance of *role* (and its manifestation as phenotype) because of its inherent relevance to biodiversity. The critical value of biodiversity lies in the myriad roles (in the sense of Simpson, 1951, 1961) that organisms exhibit that make them part of complex biotic systems. This diversity is a direct result of the different morphological, chemical, and behavioral properties that organisms display. We view role broadly as the ways in which individuals interact with their environment and the total complement of expressed properties (beyond genotype) that they exhibit; it is an organism’s correspondence to the concept of ecological niche *sensu* Hutchinson (1957); an *n*-dimensional hypervolume composed of all biotic and abiotic organismal interactions.”

Species, therefore, are not only historical units; they are functional entities, and their function is directly connected to their ecological niche, as emphasized by a close relative of the Evolutionary Species Concept, the Ecological Species Concept of Van Valen (1976): a species is “a lineage (or a closely related set of lineages) which occupies an adaptive zone minimally different from that of any other lineage in its range and which evolves separately from all lineages outside its range.” Phenotypic characters are proxies for the difficult-to-define ecological niche function of a species: Simpson (1961) considered “morphological resemblances and differences” of populations to be related to roles if such differences are adaptive. Geography can also play a role in species delimitation as another proxy for “role” (Shanker et al., 2017). It is becoming more feasible to identify not only characters that are under positive selection (are adaptive), but even the small number of genes that might actually drive speciation (e.g., Choi et al., 2020).

**FIGURE 8:**
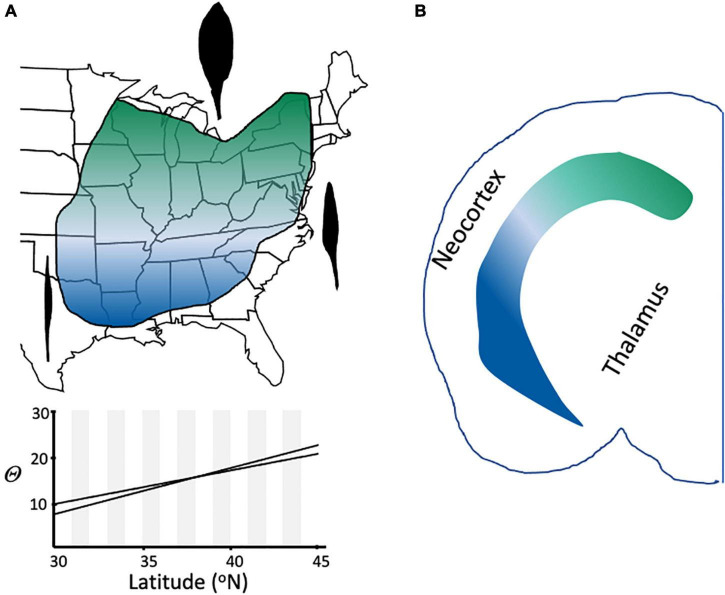
Clinal variation in species and cell types. **(A)** Leaf shape variation in *Claytonia virginica* (Doyle, 1984). Representative leaf shapes are shown as black outlines, with broad leaves being characteristic in the northern part of the range, very narrow, grasslike leaves in the extreme southern portion of the range, and intermediate shapes at intermediate latitudes in eastern North America. The 95% confidence interval of the relationship of theta (arcsin [length/width]^1/2)^ to latitude is shown for 462 individuals (*r* = 0.49; *p* < 0.001). **(B)** Continuous variation in cell type in the mouse brain (redrawn from Cembrowski and Menon, 2018). Expression of dorsal (green) and ventral (blue) marker genes for two different cell types of the hippocampus show a gradient of expression across the hippocampus.

Under the Evolutionary Species Concept, and in contrast to other species definitions (Table 1), related lineages that are geographically disjunct but share the same niche are members of the same species (Freudenstein et al., 2016). Such taxa represent one of several kinds of “cryptic species,” another being the opposite condition of taxa that are genetically and morphologically similar but have differentiated ecologically (Fiser et al., 2018). Another source of cryptic taxa is clinal variation, in which characters change gradually and continuously across the range of a species (Figure 8); clines can be caused by various phenomena, including primary divergence of populations into separate species and secondary contact between fully or partially differentiated taxa (Endler, 1977).

The role of species in ecosystems has become an increasingly important topic as the incidence of invasive species has accelerated due to climate change and globalization. One controversial topic has been whether species are interchangeable—whether it is the role they play, rather than their precise identity, that is critical for ecosystem function (Funk et al., 2017). Leuzinger and Rewald (2021) “argue that ecosystem function and ecosystem services need to be viewed not only through a taxonomic lens, but increasingly also through a functional, trait-based one.”

### Cell types

One author in the Clevers et al. (2017) opinion paper (Junhyong Kim) emphasized the cell’s “system-level roles,” noting that “A cell provides structure and it dynamically processes input environmental materials into outputs. The totality of its activity characterizes an ecological guild within the ecosystem of the organism.” He concluded that “we need an ecological definition of the cell type.” Such thinking is not new—Rowe and Stone (1977) concluded that cell type classifications should be treated as hypotheses concerning the cell’s “functional niche.” In this ecological view, each cell type is to a tissue or organ what an ecological species is to its ecosystem—each plays a particular functional role. Indeed, several other Clevers et al. (2017) authors mentioned “function” as a key criterion for defining cell type, despite the long-recognized difficulty of using function as an operational criterion (Rodieck and Brening, 1983).

Cell type classification systems prior to the availability of single cell transcriptomes were based largely on morphology, and identified a relatively small number of basic types. For plants, Xu et al. (2021) cited textbooks that list fewer than 20 cell types, most or all of which are found in more than one tissue and organ (e.g., parenchyma), though often with distinctive morphologies in different regions. For example, *Arabidopsis* leaf epidermal cells have a jigsaw puzzle shape, in contrast to the smoother borders of sepal epidermal cells (Xu et al., 2021). Epidermal cells presumably play the same general role in these and other organs, but the correlation of their morphological and spatial differences could indicate variation in their niche functions within their different cellular ecosystems. Identical transcriptomes could indicate identical functional roles, but the transcriptomes of epidermal cells in different plant organs have yet to be compared. How much difference between two transcriptomes would be required to indicate meaningfully different functions? And how does within- vs. between-region transcriptomic variance among epidermal cells compare?

Here again, the rich neuron literature provides some guidance. Variation among the vast diversity of neurons can often be categorized into a small number of groups (Stanley et al., 2020). According to Fishell and Kepecs (2020), 90% of cortical interneurons fall into four transcriptomic classes that they further subdivided into a detailed hierarchy, but they warn that this “comes with the clear caveat that both the percentage composed by these four cardinal classes and their relative contributions in specific areas will vary widely across the cortex.” Similarly, Osumi-Sutherland et al. (2021), working on a human cell atlas, noted that “cell types can be hierarchically classified and categorized in ever-increasing levels of resolution, from a general cell type such as an endothelial cell to more specialized types such as a liver sinusoidal endothelial cell (LSEC) and then down to highly specialized types found in specific locations such as a periportal LSEC.” Similar spatial fine structure also occurs in the mouse brain (Stanley et al., 2020).

Location is critical in cells as it is in real estate. Börner et al. (2021) stated that for the human cell atlas, “The spatial location of cell types within anatomical structures matters,” and Weiss et al. (2022) concluded that for melanomas “the anatomic position of the cell of origin endows it with a unique transcriptional state that makes it susceptible to only certain oncogenic insults.” But just as cell lineage and cell type can be incongruent, the same transcriptomic cell types can occur in disjunct regions (Raj et al., 2018; Yuste et al., 2020). For example, Yao et al. (2021) reported that although some of the 364 transcriptionally defined neuronal cell types they identified in the mouse isocortex were localized to very specific subregions or layers, most were much more broadly distributed. Discordance between location and cell type is not limited to vertebrates: Achim et al. (2018) reported transcriptomic vs. regional incongruence in annelid larvae. Should geographically disjunct but transcriptomically similar cells be split into different cell types? Xia and Yanai (2019) took the lumper approach: “the same cell type may be the integral building unit for many different tissues and/or organs” even though “such cell types that are found in multiple tissues and/or organs may not be exactly the same.”

For cell types as for species, function is more difficult to describe than are phenotypes that may serve as its proxies. If transcriptomes are not always well-aligned with cellular geography and environment, what about cell morphology? Peng et al. (2021) found that neurons of the mouse brain could be divided into 11 different “projection neuron types” many of which could be further subdivided into narrower morphological subgroups. Although the major types correlated with regional transcriptome variation reported by Yao et al. (2021), this was not true at the fine-grained scale, with the same transcriptomic cell types in different brain regions having different morphologies. Peng et al. (2021) concluded that “many aspects of morphological diversity cannot be accounted for by currently identified transcriptomic subtypes or clusters.” Because this morphological diversity is connected with the cell’s functional role in its specific niche in the brain ecosystem, their results highlight the need “to develop methods that enable complete reconstruction of morphology and in-depth gene-expression profiling to be conducted on the same cell” (Peng et al., 2021).

### Synthesis and questions

There are clearly parallels between a species’ role in an ecosystem and a cell’s function in a tissue, and in both cases the use of more accessible proxies for these important attributes is not foolproof. In systematics, the increasing availability of DNA sequence data has resulted in an even greater reliance on genetic lineage in defining species, but other characters clearly are also important for understanding species biology and evolution. This has led to what has been called “integrative taxonomy” (Padial et al., 2010; Fujita et al., 2012), which operationally is often an “iterative taxonomy” approach that begins with genetic lineage-based hypotheses and refines these using characters such as morphology (Yeates et al., 2011). If genetic lineages are insufficient to define species (Freudenstein et al., 2016; Sukumaran et al., 2021), then other characters must play a role analogous to supervised clustering of transcriptomic data. Ideally, such characters should be related to the ecological role of species, at least under the Evolutionary Species Concept. In cell biology, can integration of various criteria, such as transcriptome, morphology, physiology, and tissue/organ geography and ecology, be used to define and classify cell types, as envisioned by Peng et al. (2021)?

## Conclusion: Community attitudes to definitional differences

Although metaphors are never perfect, there are many parallels between the problems of defining cell types and species (Figure 1), and this suggests that there may be lessons that the two very different communities could learn from each other. The species issue is the older of the two, and there is much more literature on the subject—indeed, one of the few areas of agreement on the topic is the disclaimer, in most species concept papers, that the literature is too voluminous to review comprehensively. Both fields have been revolutionized by the ready availability of nucleotide sequence data, and modeling that reflects growing understanding of evolutionary and developmental processes is a priority in both fields. The application of explicit models to the species problem seems more advanced than comparable efforts in cell biology.

There is at least one area where cell biology seems, to this systematist, to have the advantage. One of the contributors to the Clevers et al. (2017) survey of cell biologists’ definitions of “cell type” (Joshua Sanes) drew an optimistic conclusion from the cell types as species metaphor: “Like my quarrelsome colleagues, systematists continue to debate about how to define species and even whether they exist, but this has not stood in the way of their managing to preside over a successful enterprise.” I certainly agree that systematics is a successful enterprise—it is a dynamic, rapidly evolving field, whose practitioners constantly rise to the challenge of integrating theory and data across “any and all relationships” to understand patterns of diversity and the processes that generate them. But having witnessed, first-hand, acrimonious exchanges over such issues as whether or not species can be monophyletic, any implication that cell biologists are less quarrelsome than systematists does not ring true.

The area of nomenclature is illustrative. There is broad (though by no means universal) consensus among systematists that names should reflect phylogenetic relationships. But updating pre-Darwinian Linnean binomial nomenclature based on the avalanche of molecular phylogenetic and phylogenomic results is not a simple matter. Efforts from within the systematics community to produce an explicitly phylogenetic alternative to Linnean naming rules, called the *Phylocode*^1^, were met with hostility by many systematists, generating a war of words that was quite fierce a decade or more ago, and is still capable of eliciting vitriolic reactions (Brower, 2020).

Even within the Linnean system, changes to familiar names are rarely welcome, and can be annoying even to other systematists who accept the scientific principles involved. Some evolutionary biologists believe that nomenclatural instability is more than an inconvenience. Garnett and Christidis (2017) expressed concern that the lack of uniformity in defining species—what they called “taxonomic anarchy”—hampers conservation efforts, and recommended creating a global body legally empowered to enforce a uniform process of species recognition and naming. Needless to say, the idea of involving lawyers in taxonomy and nomenclature was met with considerable pushback from others in the taxonomic community—the grim specter of Stalinist genetics was even evoked (Raposo et al., 2017). The idea of subjecting nomenclatural issues to legally binding arbitration, with the goal of mandating stability, is not a new one, however. In *Nature’s* “Scientific Correspondence” of August 1986, a very annoyed non-taxonomist excoriated taxonomists for inconveniencing yeast biologists by constantly changing scientific names (Barnett, 1986). He blasted what he called the “romantic confusion of biological classification with evolutionary studies” and concluded that “Too many taxonomists appear to subscribe to this kind of sentimental codswallop.”

It seems unlikely that cell type nomenclature will generate similar levels of controversy, at least outside of cell biology. And within that field, or at least in one part of it, there seems to be much more concord than in systematics. The Yuste et al. (2020) paper titled “A community-based transcriptomics classification and nomenclature of neocortical cell types” includes authors (Arendt, Sanes, Zeng) representing both of what I have cited as philosophically and operationally divergent approaches to the problem. Perhaps systematists should take a page from cell biology!

## Author contributions

JD conceived of and wrote the manuscript.

## Footnotes

1
http://phylonames.org/code/
